# Mycorrhizal Colonization of Wheat by Intact Extraradical Mycelium of Mn-Tolerant Native Plants Induces Different Biochemical Mechanisms of Protection

**DOI:** 10.3390/plants12112091

**Published:** 2023-05-24

**Authors:** Jorge M. S. Faria, Pedro Barrulas, Ana Paula Pinto, Isabel Brito, Dora Martins Teixeira

**Affiliations:** 1 National Institute for Agrarian and Veterinary Research, I.P. (INIAV, I.P.), Quinta do Marquês, 2780-159 Oeiras, Portugal; 2 MED, Mediterranean Institute for Agriculture, Environment and Development and CHANGE—Global Change and Sustainability Institute, Institute for Advanced Studies and Research, Évora University, Pólo da Mitra, Ap. 94, 7006-554 Évora, Portugal; app@uevora.pt (A.P.P.); ibrito@uevora.pt (I.B.); 3 HERCULES Laboratory, Évora University, Largo Marquês de Marialva 8, 7000-809 Évora, Portugal; pbarrulas@uevora.pt (P.B.); dmt@uevora.pt (D.M.T.); 4 School of Science and Technology, Évora University, Rua Romão Ramalho n°59, 7000-671 Évora, Portugal

**Keywords:** arbuscular mycorrhizal fungi, element subcellular distribution, guaiacol peroxidase, manganese toxicity, Mn-superoxide dismutase, sustainable land restoration

## Abstract

Soil with excess Mn induces toxicity and impairs crop growth. However, with the development in the soil of an intact extraradical mycelia (ERM) from arbuscular mycorrhizal fungi (AMF) symbiotic to native Mn-tolerant plants, wheat growth is promoted due to a stronger AMF colonization and subsequent increased protection against Mn toxicity. To determine the biochemical mechanisms of protection induced by this native ERM under Mn toxicity, wheat grown in soil from previously developed *Lolium rigidum* (LOL) or *Ornithopus compressus* (ORN), both strongly mycotrophic plants, was compared to wheat grown in soil from previously developed *Silene gallica* (SIL), a non-mycotrophic plant. Wheat grown after LOL or ORN had 60% higher dry weight, ca. two-fold lower Mn levels and almost double P contents. Mn in the shoots was preferentially translocated to the apoplast along with Mg and P. The activity of catalase increased; however, guaiacol peroxidase (GPX) and superoxide dismutase (SOD) showed lower activities. Wheat grown after ORN differed from that grown after LOL by displaying slightly higher Mn levels, higher root Mg and Ca levels and higher GPX and Mn-SOD activities. The AMF consortia established from these native plants can promote distinct biochemical mechanisms for protecting wheat against Mn toxicity.

## 1. Introduction

Manganese is an important micronutrient with essential functions for plant development. This element is involved in several key metabolic processes, e.g., in respiration, photosynthesis, the activation of phytohormones, aminoacid biosynthesis and the assimilation of nitrate. In addition to being an integral part of the water-splitting system of photosystem II (PSII) and RuBP carboxylase reactions, Mn plays a role in the biosynthesis of ATP, chlorophyll, isoprenoids, proteins, fatty acids, acyl lipids and secondary products; and acts as cofactor of several vital enzymes in plants; e.g., Mn-superoxide dismutase, Mn-catalase, pyruvate carboxylase and phosphoenolpyruvate carboxykinase [1].

An excess of Mn in the soil is generally a result of anthropogenic factors, such as soil contamination, or natural conditions, such as soil acidity. Although dependent on the soil’s chemical composition and edaphic properties, Mn excess compromises plant development and survival. High levels of bioavailable Mn in the soil generally lead to excessive plant internal concentrations and/or imbalances in other important nutrients [1,2]. In fact, plant uptake of Mn is not believed to be tightly controlled under excess conditions due to the existence of several transmembrane proteins capable of transporting this element [2,3]. In this way, Mn at excessive levels can compete with elements of a similar atomic radius, e.g., Ca, Co, Cu, Mg or Zn, for transmembrane transporters or metal-dependent active sites in enzymes [3]. The displacement of these elements with Mn can lead to impaired enzyme activity, metabolic alterations and macromolecular damage that induces an excessive production of reactive oxygen species (ROS) and causes oxidative stress in susceptible plants. In many plants, tolerance to Mn toxicity generally relies on the presence of a strong antioxidant response, generally carried out by enzymatic, e.g., superoxide dismutase (SOD), catalase (CAT), ascorbate peroxidase (APX) and guaiacol peroxidase (GPX); and/or non-enzymatic processes, e.g., metallothioneins, phytochelatins and the ascorbate-glutathione cycle [4,5]. Furthermore, light-dependent photosynthetic mechanisms, namely, the electron transport systems, can produce hydrogen peroxide and superoxide radicals that lead to the additional production of hydroxyl radicals in the presence of excessive levels of transition metals, such as Mn, through Fenton-type reactions [6]. Ultimately, external symptoms of Mn toxicity are impaired growth and development, leaf interveinal and marginal chlorosis, as well as leaf tip burn and necrotic spots [1,7].

In agricultural soils, Mn toxicity is commonly countered with the application of soil correctives, e.g., calcitic (Ca carbonates and oxides) or dolomitic lime (Ca and Mg carbonates). These chemical additives raise the soil’s pH and increase its levels of Ca and/or Mg, which compete with Mn for shared transporters and lower Mn toxicity in the plant. However, these correctives alter the soil’s biochemical homeostasis and are seldom applied in the correct amounts, which increases production costs for farmers [8]. In recent years, sustainable alternatives have been examined based on the use of plant growth-promoting microorganisms. Many microbial taxa, mainly fungi and bacteria, have the ability to colonize roots and provide their hosts with physiological benefits that result in increased growth and protection against biotic and abiotic stress [9]. Arbuscular mycorrhizal fungi (AMF) are responsible for the oldest and most spread mutualistic association between plants and soil microbes, and are believed to span back to the colonization of land by the first ancestors of modern plants [10]. In these symbioses, plants benefit from privileged access to water and soil nutrients, e.g., P and N, while AMF are supplied by the plant with photosynthetic carbon and other essential compounds [11]. Ecologically, AMF symbioses are responsible for shaping soil texture and fertility, increasing soil organic matter and plant resilience to abiotic and biotic stressors [12].

In soils with toxic levels of Mn, in the southern region of Portugal, the endemic AMF communities associated with native host plants appear to provide additional protection against excessive metal levels [13,14,15]. An intact ERM established by native *Lolium rigidum* (LOL) or *Ornithopus compressus* (ORN), formed from AMF consortia that are fully adapted to the soil’s stressful conditions, was seen to provide stronger colonization and protection against Mn toxicity in succeeding cultivated plants [13,14,15,16]. Surprisingly, the mechanisms responsible for this protection are dependent on the diversity of the AMF consortium previously formed, and provide different levels of protection, either by promoting stress-countering biological processes or stimulating host growth [17,18,19]. Although the taxonomic profiling of these native AMF consortia has been performed, as well as mapping their influence on wheat transcriptome [17,18,19], not much is known about the specific biochemical phenotypes they can induce in the plant host.

In the present work, this functional diversity was studied on wheat colonized by AMF consortia associated with the native plants LOL and ORN, responsible for distinct protective mechanisms, in soil with toxic levels of Mn. Wheat shoot and root growth; Mn, Mg, P and Ca levels; their respective tissue and subcellular distributions and shoot antioxidant enzyme activity were determined in order to profile some of the biochemical mechanisms induced by different AMF consortia, under Mn toxicity. Identifying the protective properties induced by AMF communities that are adapted to Mn stress contributes to the optimization of cropping systems. Selected agronomic practices based on crop sequence and no-tillage systems that keep the ERM intact, can be conducive to the enhanced functioning of indigenous microbes against biotic and abiotic stresses [20], taking advantage of natural biodiversity in the agroecosystem.

## 2. Results

### 2.1. Wheat Growth in Mn-Spiked Soil

**Figure 1:**
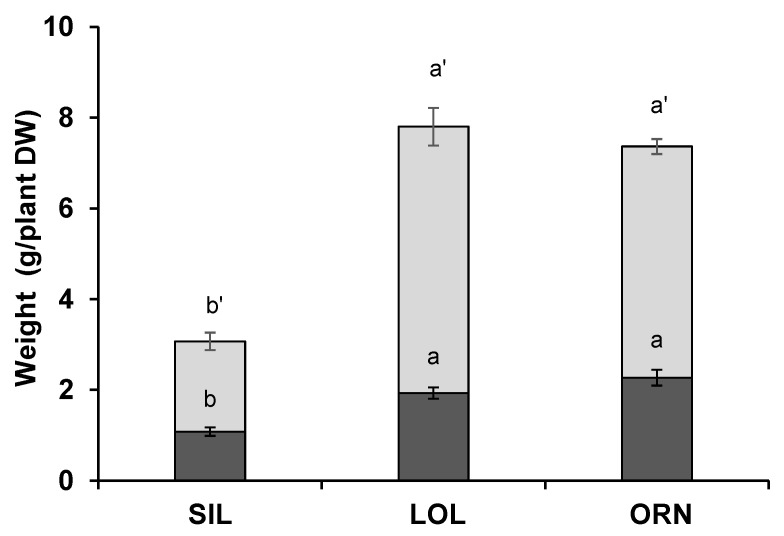
Wheat growth assessed by root (dark grey) and shoot (light grey) weight [g/plant dry weight (DW)] after 3 weeks in Mn-toxic soil where *Silene gallica* L. (SIL), *Lolium rigidum* Gaudin (LOL) or *Ornithopus compressus* L. (ORN) were previously grown. Data are presented as means ± standard error of four independent biological replicates. Different letters indicate statistically significant differences (*p* < 0.05).

Growth strongly varied between wheat grown in Mn-toxic soils with or without intact ERM (Figure 1). Wheat plantlets grown in soil from the SIL treatment showed significantly (*p* < 0.05) less weight (ca. 60%) than wheat grown in soil where a previous ERM was formed (LOL or ORN treatment) (Figure 1). The roots of wheat grown after LOL and ORN showed a 1.8- and 2.1-fold higher dry weight (DW), respectively, than those grown after SIL, while the shoot tissues showed 2.6- and 2.9-fold higher DWs, respectively (Figure 1). Additionally, no significant differences were found between the DW of wheat grown after LOL or ORN.

### 2.2. Wheat Element Levels

#### 2.2.1. Root and Shoot Mn, Mg, P and Ca Concentrations

**Figure 2:**
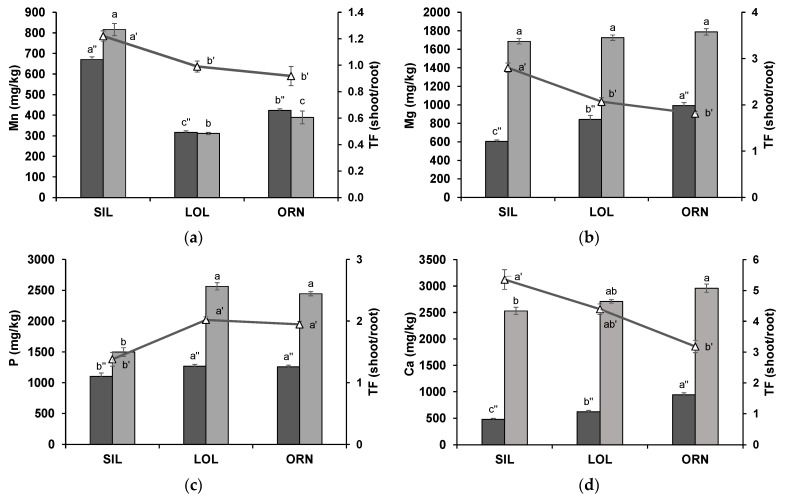
Manganese (Mn) (**a**), magnesium (Mg) (**b**), phosphorus (P) (**c**) and calcium (Ca) (**d**) concentrations [mg/kg plant dry weight (DW)] in wheat roots (dark grey) and shoots (light grey) after 3 weeks in soil where *Silene gallica* L. (SIL), *Lolium rigidum* Gaudin (LOL) or *Ornithopus compressus* L. (ORN) were previously grown. The translocation factor (TF) values are also presented (triangles). Data are presented as means ± standard error of four independent biological replicates. Different letters indicate statistically significant differences (*p* < 0.05).

The concentration of Mn was significantly higher (*p* < 0.05) in wheat grown after SIL than in wheat grown after LOL or ORN (Figure 2a). When grown in soil with an intact ERM from a native mycotrophic plant, wheat accumulated less Mn in root and shoot tissues. When compared to SIL treatment, wheat roots showed 2.1- and 1.6-fold less Mn, in LOL and ORN treatments, respectively, while the shoots showed 2.6- and 2.1-fold less Mn in the LOL and ORN treatments, respectively (Figure 2a). The translocation factor (TF) values determined for wheat grown after SIL were higher than those of wheat grown after LOL or ORN (Figure 2a), suggesting a preferential translocation of Mn to the shoots in wheat grown in soil with an intact ERM from native mycotrophic plants.

Magnesium concentrations were lower in wheat roots than in shoot tissues regardless of the treatment (Figure 2b). While no statistical differences were detected in shoot Mg concentrations between treatments, in wheat roots, Mg was found in higher concentrations in wheat grown after ORN than in wheat grown after LOL, with wheat grown after SIL showing the lowest value (Figure 2b). This tendency was also reflected in the TF values, with wheat grown after LOL or ORN showing lower values than wheat grown after SIL (Figure 2b), suggesting a lower translocation of Mg to the shoots.

Wheat grown after SIL contained approximately 40% less (*p* < 0.05) P in the shoots and ca. 10% less in the roots (*p* < 0.05) than wheat grown after LOL or ORN (Figure 2c). Concomitantly, the TF values for this element were higher for wheat grown after LOL or ORN than wheat grown after SIL, indicating higher shoot P levels. Changes in this element were expected given that this is one of the major nutritional enhancements generally induced by AMF colonization.

Calcium concentrations were higher in the shoots than in the roots (Figure 2d). In the shoots of wheat grown after ORN, Ca concentration was significantly higher (*p* < 0.05) when compared to wheat grown after SIL. In the roots, Ca concentrations were higher in wheat grown after ORN, followed by wheat grown after LOL, with roots of wheat grown after SIL showing the lowest value (Figure 2d). The TF values showed an inverse tendency, with wheat grown after ORN showing lower values, followed by LOL and SIL treatments (Figure 2d), suggesting a lower translocation of Ca to the shoots of wheat grown after ORN.

#### 2.2.2. Root and Shoot Mn, Mg, P and Ca Subcellular Distribution

**Figure 3:**
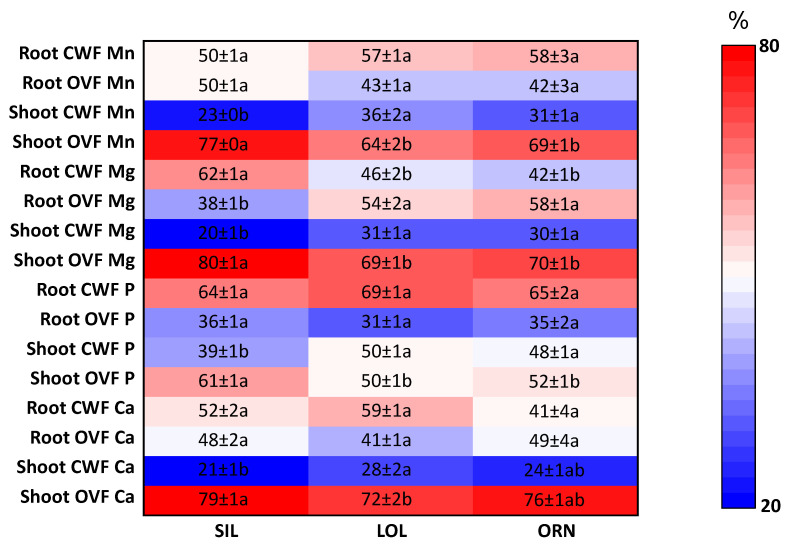
Heatmap depicting changes in subcellular partitioning (CWF, cell wall fraction; OVF, organelles and vacuole contents fraction) of Mn, Mg, P and Ca (%) in roots and shoots of wheat grown for 3 weeks in soil from previously grown *Silene gallica* L. (SIL), *Lolium rigidum* Gaudin (LOL) and *Ornithopus compressus* L. (ORN). Data are presented as means ± standard error of four independent biological replicates. Different letters indicate statistically significant differences (*p* < 0.05).

Element subcellular distribution varied in wheat grown in soil from mycotrophic native plants (LOL or ORN) when compared with wheat grown in soil after non-mycotrophic SIL (Figure 3).

Manganese was significantly higher (*p* < 0.05) in the CWF of shoots of wheat grown after LOL or ORN than in those of wheat grown after SIL. In the root system, Mn was slightly higher in the CWF in wheat grown after LOL or ORN than in those of the SIL treatment (not statistically significant).

Magnesium in wheat shoots followed the same tendency as Mn, but not in the root system. Magnesium was significantly lower (*p* < 0.05) in the OVF of shoots of wheat grown in the soil after LOL or ORN than in the shoots of wheat grown in the soil after SIL. Inversely, in wheat roots, Mg was significantly higher (*p* < 0.05) in the OVF of wheat grown after LOL or ORN than in wheat grown after SIL.

For P, although no significant changes were detected in the root system, higher proportions (*p* < 0.05) were detected in the CWF of shoots of wheat grown after LOL or ORN (ca. 1:1 proportions, CWF:OVF) than in the shoots of wheat grown after SIL (ca. 2:3 proportion, CWF:OVF).

Similar to P, Ca showed no statistically significant changes in the wheat root system, while in the shoots, Ca was significantly higher (*p* < 0.05) in the CWF of wheat grown in soil from the LOL treatment than from the SIL treatment.

### 2.3. Shoot Antioxidant Enzyme Activity

**Figure 4:**
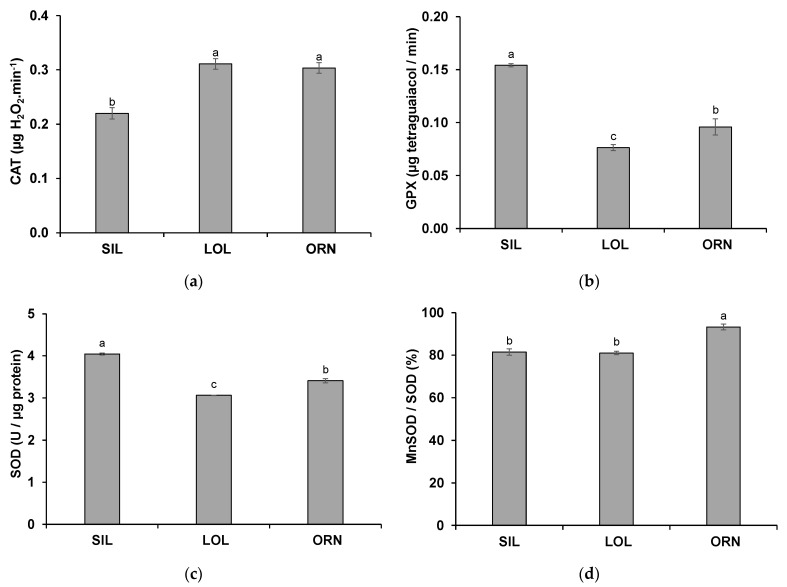
Reaction rate of catalase (CAT) (**a**) and guaiacol peroxidase (GPX) (**b**); activity of SOD (**c**) and percentage of the activity of Mn-SOD over that of total SOD (**d**) in shoots of wheat grown for 3 weeks in soil from previously grown *Silene gallica* L. (SIL), *Lolium rigidum* Gaudin (LOL) and *Ornithopus compressus* L. (ORN). Data are presented as means ± standard error of four independent biological replicates. Different letters indicate statistically significant differences (*p* < 0.05).

In shoots of wheat grown in the different soil treatments, significant differences between the activity of antioxidant enzymes were detected for CAT, GPX, SOD and Mn-SOD (Figure 4). The reaction rate of CAT was lower (*p* < 0.05) in shoots of wheat grown after SIL than in those of wheat grown after LOL or ORN (Figure 4a).

The reaction rate of GPX was higher (*p* < 0.05) in shoot extracts of wheat grown after SIL, followed by those of wheat grown after ORN, and the lowest values were obtained for shoot extracts of wheat grown after LOL (Figure 4b).

SOD activity was highest (*p* < 0.05) in shoots of wheat grown after SIL, followed by those of wheat grown after ORN, and finally, by those of wheat grown after LOL (Figure 4c). However, the ratio of Mn-SOD/total SOD was higher in shoots of wheat grown after ORN than in those of wheat grown after SIL or LOL (Figure 4d).

## 3. Discussion

Plants use a wide range of biochemical mechanisms to manage Mn toxicity, through avoidance or tolerance, which mainly depend on the plant’s species or variety. In wheat, Mn excess appears to be partly managed at the soil/root interface, likely being distributed around its roots and chelated by plant-released carboxylates [21,22]. Within wheat tissues, Mn is mostly diverted to the shoot, where its low mobility leads to an accumulation in the older leaves, easily noticeable by the induced leaf chlorosis [7,23,24]. In wheat shoots, excess Mn seems to be deposited in the vacuole, along with Mg and P, which suggests the formation of metal-phosphate complexes [25].

In the present study, the development of an ERM network by previously grown mycotrophic native plants (known to stimulate a stronger AMF colonization in a shorter amount of time, when kept intact) appears to influence the mechanisms used by wheat to counter toxic Mn levels, leading to increased wheat growth. In the soil where this ERM network was established (by LOL or ORN), wheat roots reached a higher weight, accumulated less Mn and increased the concentrations of the macronutrients Mg, P and Ca. Accordingly, the Mg/Mn ratio, a known indicator for Mn toxicity, was higher in the roots of these wheat plants, suggesting lower stress levels due to toxic Mn and, consequently, a lower probability for Mn to replace Mg at the active sites of proteins in vital biochemical pathways [1,26,27]. Inside wheat root tissues, Mn was found in higher proportions at the apoplast than at the symplast when compared to wheat grown in soil with the previous growth of a non-mycotrophic plant (SIL), while Mg was detected in higher proportions at the symplast. Changes in the subcellular partitioning of P or Ca at the roots showed no statistical significance in this study, which suggests that these elements were distributed independently of the AMF inoculum source that colonized wheat. However, it must be noted that spiking with MnCl_2_ can slightly alter soil pH and cation exchange capacity (CEC), leading to higher levels of Ca^2+^ in soil solution, which can influence plant Ca levels [28].

The roots of mycorrhizal plants are composed of their plant and fungal partners. For wheat grown in the soil from the two types of native plants tested, mycorrhizal colonization was extensive. In previous studies, the AMF colonization of wheat roots, measured by the magnified intersections method, was only 23% for wheat grown in the soil after SIL; however, in the soil where LOL or ORN were previously grown, this value ascended to 50 and 56%, respectively, indicating a stronger AMF colonization [13]. Consequently, this increase in fungal matter must be considered when analyzing the changes in Mn and Mg. Indeed, under Mn excess, some AMF isolates appear to act as a buffer and retain Mn in their structures, partly explaining the lower Mn levels of the host [29]. Additionally, AMF are known to influence the soil microbiome, managing the communities of Mn-oxidizing and Mn-reducing bacteria, which directly influence the bioavailability of toxic Mn for plant uptake [30,31,32]. Magnesium has also been linked to the alleviation of Mn toxicity. In experiments where Mg was added to maize root segments colonized with *Glomus claroideum*, under Mn toxic conditions, hyphal growth was enhanced, and maize biomass increased [33].

In the present work, the presence of a pre-established ERM network in the soil promoted a higher growth of wheat shoots than the roots; i.e., a higher shoot/root ratio. In wheat grown in these soils, shoot Mn concentrations were lower, while Mg concentrations showed no significant changes. At the subcellular level, Mn was redirected to the apoplast along with Mg. As expected, P concentrations were greatly increased in shoot tissues, showing a mostly even subcellular distribution. In contrast, in wheat grown after SIL, a 2:3 partition (apoplast:symplast) was observed. Calcium was also increased, but it was only statistically significant in wheat grown after ORN; however, significant changes in subcellular partitioning were only detected for shoots of wheat grown after LOL, with Ca being relocated to the apoplast.

The accumulation of P in mycorrhizal plants is a well-known benefit of AMF colonization and appears to be ubiquitous, with some exceptions. However, other nutrients have shown different responses. For example, in switchgrass, several AMF species were tested for their ability to alter shoot Al, Ca, Cu, K, Mg, Mn, N, P, S and Zn levels [34,35]. While shoot P was seen to increase to different degrees irrespective of the AMF species, the other elements showed either increasing or decreasing concentrations depending on the AMF species, in comparison to non-colonized switchgrass. Under Mn toxicity, this effect was also seen in sorghum colonized by different AMF isolates [36]. The levels of Ca, Cu, Fe, K, Mg, S and Zn were differently influenced in both the roots and shoots, suggesting a functional diversity in AMF species. In the present study, the AMF consortia established in the soil by different native plants also appear to influence the biochemical mechanisms involved in coping with Mn excess in slightly different ways. Wheat grown after LOL had lower Mn concentrations than that grown after ORN; however, these accumulated higher levels of Mg and Ca in the roots.

The activity of the antioxidant enzymes from shoot extracts of wheat grown in the analyzed soil treatments also showed variations. Wheat grown in soil from the non-mycotrophic native plant SIL showed a lower CAT activity but higher GPX and SOD activities. However, soil from the mycotrophic LOL or ORN induced different responses in wheat. For wheat grown in ORN soil treatment, the activities of GPX and SOD were higher, but most notably the activity of Mn-SOD was higher than in wheat grown after SIL or LOL. This enzyme appears to be directly influenced by the AMF consortia and associated microbiome formed by ORN in the soil.

SOD isoenzymes can be classified into three groups based on their associated metalloid cofactor; iron SODs (Fe-SOD) are mainly located in the chloroplast, copper-zinc SODs (Cu/Zn-SOD), believed to have evolved later in eukaryotes, are distributed in the chloroplast, cytosol and possibly the extracellular space, while manganese SODs (Mn-SODs) are mainly found in the mitochondrion and the peroxisome [37,38]. Under excess Mn, an increase in SOD and/or Mn-SOD activity has been reported for important crops, such as tomatoes [39], rice [40,41] and cucumbers [5]. An increase in the oxidative stress-coping enzymatic mechanisms appears to be a broad response to Mn toxicity. In fact, plants with the potential for the phytoremediation of Mn-toxic soils, such as *Broussonetia papyrifera* [42], or Mn hyperaccumulators, such as *Phytolacca americana* [43,44], rely on increased SOD activity to counter the negative effects of Mn excess. In wheat, an increase in SOD activity was a major factor distinguishing tolerant from sensitive lines, with cell cultures of tolerant lines exhibiting greater SOD activities than those of sensitive lines [45,46]. Furthermore, tolerant wheat plants have been seen to resist excess Mn and show no decline in photosynthesis or respiration, and no additional production of chelator organic acids, unlike sensitive wheat lines, which suggests they can counter oxidative stress in these compartments (chloroplasts and mitochondria) [47]. In fact, in perennial ryegrass under Mn toxicity, the Mn tolerant line exhibited high Fe-SOD activities, while the sensitive line showed higher Mn- and Cu/Zn-SOD activities [4]. In the present work, the proportion of Mn-SOD activity was higher for wheat grown after ORN. This appears to be a distinguishing factor for the biochemical mechanisms involved in countering Mn toxicity in the ORN treatment, suggesting an investment in countering the oxidative stress resulting from respiration in the mitochondrial compartment [48].

Variations in the biochemical parameters between soil treatments are partly explained by the different AMF consortia and associated microbiome gathered by the previously grown native plants. In previous studies, the diversity and community structure of these consortia have been shown to be different between each native plant and were identified as determinants for the respective symbiotic interactions established with wheat [16,19]. Metagenomic analysis of LOL roots showed a lower AMF diversity than in ORN roots, with roughly half of the operational taxonomic units (OTUs) being reported. The AMF community in LOL roots had mainly *Rhizophagus* sp., unspecified Glomeraceae and *Claroideoglomus* sp.; while in ORN, roots were mainly colonized by the previously mentioned OTUs, but also by *Archaeospora* sp., *Acaulospora* sp. and *Pacispora* sp. In wheat grown after LOL or ORN, the number of OTUs reported was ca. 23. While LOL roots were favorably colonized by *Claroideoglomus* sp. and unspecified Glomeraceae, a profile which is roughly similar to the profile obtained for the roots of wheat grown in the absence of a mycotrophic native plant, in wheat grown after ORN, *Rhizophagus* sp. was the main AMF genus. Thus, in wheat, the AMF symbiosis promoted by the previous establishment of specific ERM (associated with LOL or ORN), varied in predominance and diversity, which may be responsible for the difference in its response to Mn toxicity [17,18], as well as for the changes in the biochemical mechanisms identified in the present work.

## 4. Materials and Methods

### 4.1. Soil Characterization

The tested soil was a granitic Eutric Cambisol collected from the top 20 cm of natural pasture in the vicinity of the Évora University experimental farm grounds, Alentejo, Portugal (38°32′ N; 08°00′ W). Soil fertility was assessed at the Laboratório Químico Agrícola Rebelo da Silva, of the Instituto Nacional de Investigação Agrária e Veterinária (INIAV, I.P.), Oeiras, Portugal, a certified laboratory for soil analysis. The soil (air dried and sieved to 2 mm particles) was characterized as a sandy loam soil with 67 mg K/kg (Egner-Rhiem/Flame atomic emission spectroscopy), 112 mg Mg/kg (1 M ammonium acetate at pH 7, Atomic absorption spectroscopy), 41 mg Mn/kg (Lakanen/Flame atomic absorption spectroscopy), 0.4 mg N-NO_3_/kg [13], 26 mg P/kg (Egner-Rhiem/UV–Vis. molecular absorption spectrophotometry), soil organic matter (SOM) at 11 g/kg (chromic acid wet oxidation), a cation exchange capacity (CEC) of 4.5 centimoles of charge per kg [cmol(+)/kg], a base saturation of 60% and a pH of 5.6 [soil:water = 1:2.5 (*w*/*v*)]. For this soil, the abundance of AMF was previously quantified at 180 viable propagules per g of dry weight [13]. The specific diversity of the AMF community associated with the roots of LOL or ORN and wheat roots after each native plant has been previously described for this system [19]. Soil spiking with Mn was performed by adding an MnCl_2_ solution [15 mg/kg soil dry weight (DW)] and manually mixing the soil. The homogenized Mn toxic soil was left to stabilize in 8 L pots for 1 week, with soil hydration maintained at approximately 70% of maximum water holding capacity, by weight [23,49].

### 4.2. Plant Material and Experimental Protocol

Experiments were performed at the greenhouse complex of the Évora University experimental farm grounds, Alentejo, Portugal. Plant material for biochemical profiling was kindly provided by the Mediterranean Institute for Agriculture, Environment and Development (MED). For the greenhouse experiments, seeds of *Silene gallica* L. (SIL), *Lolium rigidum* Gaudin (LOL) and *Ornithopus compressus* L. (ORN) were collected from naturally growing populations in the field and *Triticum aestivum* L. (cv. Ardilla) was acquired from local certified sellers. Viable seedlings were obtained by germinating seeds on hydrated filter paper covered with a transparent film to avoid desiccation. To obtain the specific soil microbiological environment characteristic of each developer plant in the Mn-toxic soil, seedlings of SIL (a non-mycotrophic plant that does not develop ERM), LOL or ORN (both highly mycotrophic plants that develop an intricate ERM network) were planted in 8 L dark plastic pots and left to grow for seven weeks. During this time, plants developed their specific symbiotic microbiological communities and associated arbuscular ERM. Four pots per developer species, with five equally distanced seedlings each, were set up in the greenhouse and kept fully randomized throughout. The pots were weighed every day and kept hydrated to ca. 70% of the maximum water holding capacity, by weight. The air temperature (maximum and minimum) was recorded daily and kept below 30 °C by greenhouse cooling. Any wild weed species that germinated were manually removed to avoid the development of unspecific AMF consortia. After seven weeks, plant shoots were cut and kept in the soil surface. The pots were then maintained for one week in the conditions described above for soil stabilization. Subsequently, six equally distanced wheat seedlings were planted in each pot and left to grow for three weeks for each soil treatment (four replicate pots). At the end of this growth period, wheat plants (four replicates) were collected, and the shoots were weighed, immediately frozen in liquid nitrogen and stored at −80 °C until analysis.

### 4.3. Subcellular Fractionation of Wheat Tissues

The apoplast portion of wheat shoots and roots (cell wall fraction (CWF), mainly composed of cell walls, cellular debris and any metal granules) was isolated from the symplast portion (with organelle components, cytoplasm and vacuole; designated as the organelles and vacuole contents fraction, OVF) through differential centrifugation [50,51]. Hence, frozen wheat shoots were ground in liquid nitrogen and homogenized in buffer solution [250 mM sucrose, 1.0 mM dithioerythritol and 50 mM Tris–HCl (pH 7.5)] with a ratio of 200 mg/5 mL buffer solution, and the homogenate was centrifuged at 2500× *g*, at 4 °C for 15 min. The pellet contained the CWF, while the supernatant contained the OVF. All fractions were frozen and kept at −80 °C until analysis.

### 4.4. Quantification of Mn, Mg, P and Ca in Wheat Tissues and Tissue Fractions

Elemental quantification was performed on the digested solutions of wheat root and shoots (50 mg) and on the respective fractions. For digestion, plant material was freeze-dried on a Telstar LyoQuest lyophilizer (Telstar, Terrassa, Spain) for three days and then pre-digested in closed Teflon beakers with 2 mL of HNO_3_ (Suprapur, 67–69%, Fisher Chemicals, Hampton, NH, USA) for 12 h. Following, solutions were digested by heating up to 120 °C, for 24 h; and 0.5 mL of H_2_O_2_ (Suprapur, 30%, Merck, KGaA, Darmstadt, Germany) was then added to further digest the organic material at 80 °C. Finally, the digested samples were heated to 100 °C and the remaining solid residue was resuspended in 50 mL of a 2% HNO_3_ aqueous solution for ICP-MS analysis. The certified reference material, NIST SRM 1573a (tomato leaves), and one digestion blank were submitted to the same digestion procedure for method validation, namely, the evaluation of the accuracy, precision and limits of detection for each element. For element quantification, digested samples were analyzed with an Agilent 8800 Triple Quadrupole ICP-MS equipped with a Micromist nebulizer, according to a previously reported methodology [14]. The collision/reaction cell was set to “no-gas mode” for the quantification of Mn and Mg, “O_2_ mode” for the quantification of P and “NH_3_ mode” for the quantification of Ca. The plasma gas flow rate was 15 mL/min, and the collision and reaction gas flow rate was 0.5 mL/min for O_2_. Analyses were optimized at 1550 W forward power and a 1.1 L/min Ar carrier gas flow with no dilution or makeup gas. Sampling depth (10 mm) and lens parameters were optimized for the highest sensitivity and optimum peak shape while maintaining low oxides and doubly charged species. An MS/MS scan type was used in all the operation modes.

### 4.5. Antioxidant Enzymatic Activity

The protein extracts used for the determination of antioxidant enzyme activities were obtained by centrifuging the homogenate of ground flash frozen samples of wheat shoots (50 mg) in 1 mL of 50 mM potassium phosphate buffer (pH 7.0) at 12,000× *g* and 4 °C for 20 min [52]. The supernatant was immediately used in the protein quantification and measurement of the activity of CAT, GPX, SOD and Mn-SOD. Absorbances of four experimental replicates were recorded in a Thermo Scientific™ Multiskan™ Microplate Spectrophotometer, each with three instrumental replicates. The protein content was determined against a bovine serum albumin (BSA) calibration curve using Bradford reagent [53]. CAT activity was assessed by following H_2_O_2_ consumption at 240 nm for 3 min and determining enzyme activity as µg H_2_O_2_ decomposed/min/mg of protein, using an extinction coefficient of 39.4 L/mol cm [54]. GPX activity was assessed by following the formation of guaiacol tetramer (tetraguaiacol) at 470 nm for 2 min and determining enzyme activity as µg tetraguaiacol formed/min/mg of protein, using an extinction coefficient of 26.6 L/mmol cm [55]. SOD activity was determined according to Beyer and Fridovich [56]. All reagents were kept under dark conditions, and the reactions were duplicated, with one group maintained under the dark and the other exposed to light (15 W) for 15 min. The SOD inhibition of formazan formation was determined at 560 nm and one SOD activity unit (U) corresponded to the enzyme activity required to inhibit NBT photoreduction by 50%. Mn-SOD was determined using the SOD activity methodology described above with the exception that the protein extract was initially incubated for 30 min in 10 mM H_2_O_2_ to inhibit Fe- and Cu/Zn-SODs [41]. SOD and Mn-SOD activities were expressed as U/mg protein.

### 4.6. Data Treatment and Statistical Analysis

The data obtained from ICP-MS analyses were processed with version 28 of SPSS Statistics software (IBM, New York, NY, USA). The statistical significance of the data was determined with one-way ANOVA, and individual means were compared using the Tukey’s post-hoc test with *p* < 0.05; the Shapiro–Wilk Test ensured data normality and the Browns–Forsythe test was used for homoscedasticity [23].

The activity of antioxidant enzymes was analyzed with version 2019 of Origin Graphing and Analysis software (OriginLab, Northampton, MA, USA). For the activity of CAT and GPX, a nonlinear regression analysis was performed by plotting H_2_O_2_ consumption for CAT, or tetraguaiacol formation for GPX, against time values (in min) and by fitting a growth equation. The values obtained for the initial reaction rates (slope) were recorded. For SOD and Mn-SOD, absolute values were analyzed [48]. The statistical significance of the data was determined with one-way ANOVA as described above.

Element translocation factors (TF) were determined for each element according to the following formula [57]:

TF = C_shoot_/C_root_, where C_shoot_ and C_root_ are the element concentrations in shoot and root tissues, respectively.

## 5. Conclusions

The use of native mycotrophic plants as developers of ERM in soils affected by Mn toxicity is a sustainable strategy to promote subsequent crop growth (e.g., wheat) in soils with Mn excess. The biochemical mechanisms responsible for increased crop resilience to Mn toxicity, as a result of colonization by intact ERM, appear to differ according to the AMF fungal community associated with the native ERM developer plant. In wheat, lower Mn levels and higher concentrations of P and Ca were generally a response to soil with these intact ERM. However, a higher activity of Mn-SOD, a SOD isozyme located at the mitochondria and peroxisomes, was preferably triggered by the AMF symbiosis established between wheat and the ERM previously developed by native *O. compressus*, probably providing protection against the oxidative stress induced by Mn excess on the electron transport chain and the biochemical reactions of respiration.

## Data Availability

The raw data supporting the findings of this study are available from the corresponding author (Jorge M. S. Faria) upon reasonable request.
